# Regulation of primary plant metabolism during plant-pathogen interactions and its contribution to plant defense

**DOI:** 10.3389/fpls.2014.00017

**Published:** 2014-02-10

**Authors:** Clemencia M. Rojas, Muthappa Senthil-Kumar, Vered Tzin, Kirankumar S. Mysore

**Affiliations:** ^1^Plant Biology Division, The Samuel Roberts Noble FoundationArdmore, OK, USA; ^2^National Institute of Plant Genome Research, Jawaharlal Nehru University CampusNew Delhi, India; ^3^Boyce Thompson Institute for Plant ResearchIthaca, NY, USA

**Keywords:** primary metabolism, plant defense, virulent pathogens, avirulent pathogens, programmed cell death, hypersensitive response

## Abstract

Plants are constantly exposed to microorganisms in the environment and, as a result, have evolved intricate mechanisms to recognize and defend themselves against potential pathogens. One of these responses is the downregulation of photosynthesis and other processes associated with primary metabolism that are essential for plant growth. It has been suggested that the energy saved by downregulation of primary metabolism is diverted and used for defense responses. However, several studies have shown that upregulation of primary metabolism also occurs during plant-pathogen interactions. We propose that upregulation of primary metabolism modulates signal transduction cascades that lead to plant defense responses. In support of this thought, we here compile evidence from the literature to show that upon exposure to pathogens or elicitors, plants induce several genes associated with primary metabolic pathways, such as those involved in the synthesis or degradation of carbohydrates, amino acids and lipids. In addition, genetic studies have confirmed the involvement of these metabolic pathways in plant defense responses. This review provides a new perspective highlighting the relevance of primary metabolism in regulating plant defense against pathogens with the hope to stimulate further research in this area.

## INTRODUCTION

Plants rely on innate immunity to protect themselves from the threats of pathogens. Such innate immunity is based on preformed and induced defense responses (Mysore and Ryu, 2004). Preformed defense responses are nonspecific and include compounds with antimicrobial properties or structural barriers such as the cell wall and the cytoskeleton that deter pathogens and pests (Mysore and Ryu, 2004; Senthil-Kumar and Mysore, 2013). Induced defenses are activated by the recognition of pathogen-associated molecular patterns (PAMPs) present on the pathogen surface (Boller and He, 2009) or by recognition of proteins (effectors) translocated by the pathogen to the host cell (Jones and Dangl, 2006; Bonardi and Dangl, 2012). Early induced defense responses include cytoskeletal reorganization (Hardham et al., 2007; Higaki et al., 2011), cell wall fortification (Hardham et al., 2007), generation of reactive oxygen species (ROS; Torres, 2010), and synthesis of phytoalexins (Ahuja et al., 2012), while later events during defense responses include transcription of pathogenesis-related (PR) proteins (van Loon et al., 2006) and the development of a type of programmed cell death (PCD) known as the hypersensitive response (HR) that limit pathogen spread (Coll et al., 2011). Although considerable progress has been made to understand plant defense responses, very little is known about the role of primary metabolic pathways required for growth and development in regulating plant defense responses.

For many years, it has been suggested that the role of primary metabolism during plant-pathogen interactions is to support cellular energy requirements for plant defense responses (Bolton, 2009; Kangasjarvi et al., 2012). Energy is critical during the execution of plant defense responses due to the expression of hundreds of genes from multiple defense pathways (Scheideler et al., 2002). In addition, defense responses appear to impose a fitness cost; *Arabidopsis* mutant plants that constitutively express defense responses are stunted and have reduced fertility while mutant plants defective in defense signaling pathways are taller (Heil and Baldwin, 2002). Therefore, it appears that in order to establish a favorable energy balance for defense, the upregulation of defense-related pathways is compensated by the downregulation of genes involved in other metabolic pathways. Consistent with this notion, genes involved in photosynthesis and chlorophyll biosynthesis have been found to be downregulated upon challenge by virulent and avirulent pathogens as well as pathogen-derived elicitors (Scholes and Rolfe, 1996; Ehness et al., 1997; Mouly et al., 1998; Berger et al., 2004; Swarbrick et al., 2006; Truman et al., 2006; Denoux et al., 2008; Bilgin et al., 2010). Interestingly, when using chlorophyll fluorescence imaging in different plant-microbe interactions, it was reported that changes in photosynthesis occurred locally at the infection site and the tissue immediately surrounding it (Berger et al., 2004; Scharte et al., 2005; Bonfig et al., 2006), and the decrease in photosynthesis was faster and more pronounced after inoculation with an avirulent strain (Scharte et al., 2005; Bonfig et al., 2006). Although light reactions during photosynthesis generate chloroplastic ROS which could be used for defense responses, downregulation of photosynthesis is counterintuitive (Zeier et al., 2004; Zurbriggen et al., 2009), and no experimental evidence is available to explain why it happens. Nevertheless, two possible mechanisms have been proposed: (1) suppression of photosynthesis triggered by pathogen effectors (Truman et al., 2006) and (2) feedback regulation mediated by sugar signals (Herbers et al., 1996a; Scharte et al., 2005; Rolland et al., 2006). Regardless of the mechanism, downregulation of photosynthesis likely alleviates the energy expenditure associated with the upregulation of other pathways that provide such energy. For example, energy can be derived by increasing the activities of respiratory metabolism, cell wall invertase and carbohydrate transporters (Scharte et al., 2005; Essmann et al., 2008). Such a metabolic shift from source to sink may further enhance the expression of defense-related genes and the production of plant secondary metabolites such as phytoalexins (Bolton, 2009).

While the role of primary metabolism as energy provider is undeniable, this review focuses mainly on the role of primary metabolism regulating defense responses in plants in the presence of potential pathogens or pathogen-derived elicitors. In order to show a clear picture regarding the function of primary metabolism in defense, we have selected examples from the literature that highlight plant responses associated with avirulent pathogens (those that induce defense responses without causing disease) or triggered by pathogen-derived elicitors (purified molecules that induce defense responses), such as the well characterized Flg22 and HrpZ (Preston et al., 1995; Felix et al., 1999). Although most of these responses also occur with virulent pathogens [that effectively cause disease (Alfano and Collmer, 1996)], we won’t discuss them extensively because the pathogen itself determines the outcome in those cases, and it is difficult to assess the host contribution. Extensive research has demonstrated that bacterial pathogen effector proteins translocated via specialized secretion machinery (e.g., the type III secretion system) suppress plant basal defense responses activated upon PAMP perception, and, in some instances, specific bacterial effectors modify plant metabolism to thrive in the plant environment (Truman et al., 2006). Several reviews have already covered this topic (Espinosa and Alfano, 2004; Mudgett, 2005; Rico et al., 2011).

## PLANT PRIMARY METABOLIC PATHWAYS AND THEIR ROLE IN PLANT DEFENSE

The association between primary metabolism and defense responses has been drawn from expression analysis of genes encoding transcription factors and metabolic enzymes upon exposure of *Arabidopsis* plants to biotic stresses such as the virulent pathogen *Phytophthora infestans *and the avirulent pathogens *Pseudomonas syringae *pv. tomato DC3000 (Hrc^-^) and *P. syringae *pv. tomato DC3000 (*AvrRpm1*), and the exposure to pathogen-derived elicitors Flg22 and HrpZ (Less et al., 2011). It was observed that after treatment with virulent or avirulent pathogens or pathogen-derived elicitors, transcripts from specific functional categories were upregulated while others were downregulated. Upregulated transcripts were associated with processes involved in energy production, such as glycolysis and the pentose phosphate pathway, TCA cycle, mitochondrial electron transport, ATP biosynthesis, and biosynthesis of some amino acids such as lysine and methionine whose catabolism leads to energy production, as well as biosynthesis of glutamic acid, arginine, serine, and glycine that are associated with photorespiration (Less et al., 2011). Downregulated genes were associated with assimilatory processes such as photosynthesis, starch metabolism, lipid metabolism, C1 metabolism and biosynthesis of amino acids leucine, isoleucine, and valine (Less et al., 2011). Before this genome-wide study, several wet-lab studies focusing on one or a few genes involved in primary metabolism had also provided hints supporting a function of primary metabolism in regulating known defense pathways. Given the complexity and the abundance of primary metabolic pathways, we chose only studies with convincing experimental evidence involving carbohydrate, amino acid and lipid metabolic pathways to support the link between primary metabolism and defense responses.

### CARBOHYDRATE METABOLISM

**FIGURE 1:**
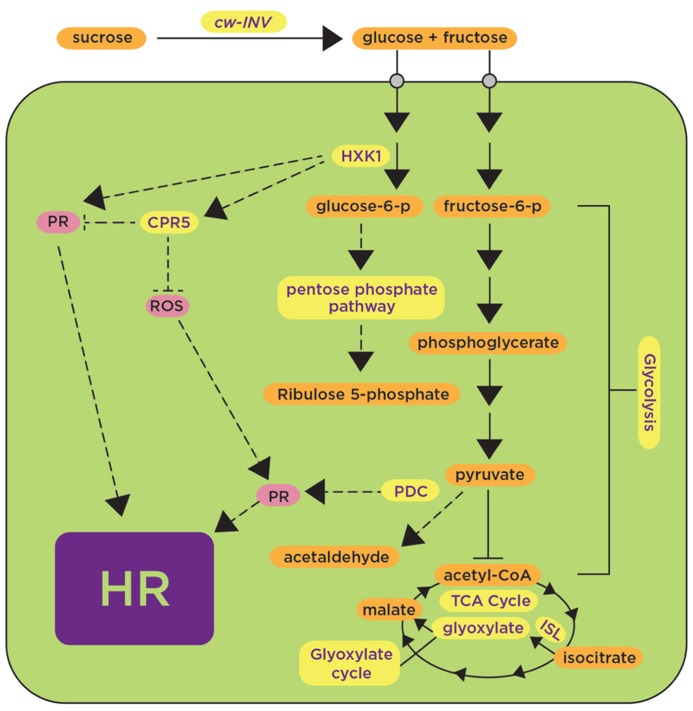
**Induction of carbohydrate metabolic pathways upon plant’s exposure to avirulent pathogens and pathogen-derived elicitors contributes to positive and negative regulation of defense responses.** Carbohydrate metabolic pathways such as glycolysis, the pentose phosphate pathway and the tricarboxylic acid cycle (TCA) are shown in yellow, indicating upregulation in the presence of avirulent pathogens or pathogen-derived elicitors. Some proteins, such as CwINV, HXK, CPR5, and PDC, are highlighted as components proposed to play a positive (arrows) or negative (blunt-ended lines) role in regulating known defense responses (shown in pink) such as the generation of reactive oxygen species (ROS) and the activation of pathogenesis-related proteins (PR) leading to the hypersensitive response (HR). HXK, hexokinase; CPR5, constitutive expresser of *PR* genes 5; PDC, pyruvate decarboxylase; ISL, isocitrate lyase. Dashed lines represent hypothetical regulatory mechanisms.

The mechanism linking carbohydrate metabolism with defense responses started to be elucidated when experiments demonstrated induction of *PR* genes by sugars in the absence of pathogens. Leaf disks of tobacco floated with glucose, fructose and sucrose solutions induced *PR* transcripts *PR-Q *and *PAR1* *photo-assimilate responding gene 1* (Herbers et al., 1996b). Other sugars have also been implicated in defense responses (Reza et al., 2012). In *Arabidopsis*, the induction of *PR-1 *and *PR-5 *by glucose was demonstrated in liquid cultures and shown to be dependent on *AtHXK1* (*hexokinase 1*; Xiao et al., 2000), that phosphorylate glucose to glucose-6-phosphate, the first step in the glucose metabolic pathway (**Figure 1**). Together, these results suggest that carbohydrate metabolism positively regulates the expression of defense-related genes. In addition to the positive regulatory function of HXK1, HXK1 might also have a negative regulatory role as evidenced from studies in *Nicotiana benthamiana* where downregulation of *HXK1 *by virus-induced gene silencing (VIGS) caused an increased accumulation of H_2_O_2_, increased expression of transcripts associated with defense responses and increased cell death, indicating that HXK1 negatively regulates PCD (Kim et al., 2006). At first glance, these studies regarding the positive and negative regulation of defense responses mediated by HXK1 are not necessarily comparable because they used different plant materials (leaf disks, cell cultures and whole plants) and different plant species (*Arabidopsis* and *N. benthamiana*); however, a previous work on AtHXK1 had revealed a dual role for it as a sugar sensor mediating both activation and repression of sugar-responsive genes (Jang et al., 1997). It is possible that in the context of defense responses, HXK1 also may have a dual regulatory role.

Another gene involved in carbohydrate metabolism is HYS1 (Hypersenescence1); the *HYS1* mutant was proposed to have altered sensitivity to sugars or altered sugar signaling, likely mediated by hexokinase (Yoshida et al., 2002). *HYS1 *is allelic to *CPR5 *(constitutive expresser of *PR* genes 5; Bowling et al., 1997; Boch et al., 1998). The *Arabidopsis* *cpr5 *mutant shows constitutive pathogen defense responses such as accumulation of ROS, expression of *PR* genes and elevated levels of salicylic acid (SA) (Bowling et al., 1997; Boch et al., 1998; **Figure 1**). *cpr5* also showed resistance against virulent pathogens *P. syringae *pv. maculicola (Bowling et al., 1997; Boch et al., 1998), *Hyaloperonospora arabidopsidis* strain Noco2 (Bowling et al., 1997), and *P. syringae *pv. tomato DC3000 (Boch et al., 1998), as demonstrated by symptom reduction and less pathogen growth in comparison with wild-type plants (Bowling et al., 1997). Moreover, expression of the *PR* gene *BGL2 *(β 1,3-glucanase) using a *BGL2-GUS *reporter gene (Cao et al., 1994) revealed 1.5-fold higher GUS activity in the untreated *Arabidopsis* *cpr5 *mutant in comparison with INA (SA-analog)-treated wild-type plants (Bowling et al., 1997). Together, all these data provide genetic evidence supporting a role of sugar metabolism regulating events associated with defense responses such as accumulation of H_2_O_2_, expression of *PR *genes, accumulation of SA and elicitation of the HR.

Further evidence for the role of carbohydrates in plant defense responses has been provided by experiments showing the induction of genes involved in carbohydrate metabolism upon challenge by pathogens or pathogen-derived elicitors. Considerable attention has been devoted to the cell wall invertase (cwINV) that cleaves sucrose into fructose and glucose. Several genes encoding this enzyme have been shown to be induced or their corresponding enzymatic activities increased in different plant species after treatment with pathogen-derived elicitors such as the fungi-derived chitosan (Ehness et al., 1997) or an avirulent pathogen [*Blumeria graminis *f. sp. *hordei *race A6 (*AvrMla12*); Swarbrick et al., 2006; **Figure 1**]. Genome-wide studies using cDNA arrays in *Arabidopsis thaliana *infected with the avirulent pathogen *P. syringae *pv. tomato (*AvrRpt2*) revealed upregulation of some transcripts from glycolysis (hexokinase and glyceraldehyde dehydrogenease), Krebs cycle (pyruvate dehydrogenase, aconitase, α-ketoglutarate dehydrogenase), oxidative pentose phosphate pathway (ribose-5-phosphate isomerase), and glyoxylate metabolism (isocitrate lyase and oxalyl-CoA dehydrogenase; Scheideler et al., 2002; **Figure 1**). As mentioned above, the accumulation of soluble carbohydrates associated with the upregulation of the genes mentioned was proposed to repress photosynthetic genes to allow the induction of defense responses (Bolton, 2009); however, it was not demonstrated how the latter would occur. More convincing evidence for the role of carbohydrates in defense responses is the resistance phenotypes observed in transgenic plants overexpressing genes involved in carbohydrate metabolism. Tobacco transgenic plants expressing the pyruvate decarboxylase (*PDC*) gene were generated to determine whether metabolic imbalance, caused by overexpression of *PDC*, triggers PCD (Tadege et al., 1998). Indeed, *PDC* overexpressors had increased levels of soluble sugars and exported sucrose, and displayed a lesion mimic phenotype with increased callose deposition and expression of *PR* genes (Tadege et al., 1998). Significantly, upon inoculation with *P. infestans*, *PDC* overexpressors impaired pathogen spread (Tadege et al., 1998). The authors suggest that altered sugar levels associated with overexpression of *PDC* are used as a signal to activate the same or a similar PCD pathway as the lesion mimic mutants do (Tadege et al., 1998; **Figure 1**). Similarly, tobacco plants expressing yeast invertase in apoplast and vacuole also developed spontaneous necrotic lesions (Herbers et al., 1996a). These plants had a strong accumulation of transcripts for *PAR-1*, as well as *PR-1b* and *PR-Q *(Herbers et al., 1996a). In addition, these plants showed increased levels of phytoalexin (capsidiol) and SA, and exhibited systemic acquired resistance (SAR; Herbers et al., 1996a). In contrast, RNAi lines generated in tobacco to downregulate *cwINV* had reduced callose deposition, less accumulation of H_2_O_2_ and reduced expression of *PR* genes, and showed reduced resistance to *Phytophthora nicotianae* in comparison with the wild-type resistant cultivar (Essmann et al., 2008).

Collectively, transcriptomics and genetics data support the general view that avirulent pathogens or pathogen-derived elicitors induce the expression of genes involved in carbohydrate metabolism processes such as glycolysis, the pentose phosphate pathway and the TCA cycle (**Figure 1**). The expression of these genes affects downstream defense responses such as the generation of ROS and the activation of *PR *genes that precedes the onset of the HR. In the case of *cwINV* and *PDC*, defense responses are induced, while in the case of *CPR5*, defense responses are repressed (**Figure 1**)*. *It appears that the regulation of sugar-mediated defense responses operates at multiple levels, but detailed characterization of potential regulatory nodes and the transcription factors involved is still needed.

### PHOTORESPIRATION

**FIGURE 2:**
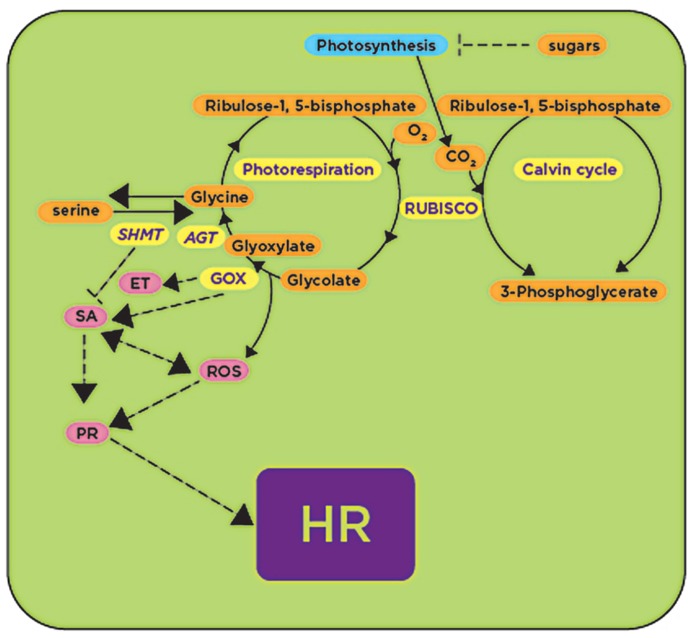
**RUBISCO and RUBISCO-mediated processes: photo-respiration and carbon fixation are upregulated upon plant’s exposure to avirulent pathogens and pathogen-derived elicitors (shown in yellow), while under the same conditions photosynthesis is downregulated (shown in blue)**. GOX activity during photorespiration generates reactive oxygen species (ROS) that activate (arrows) downstream defense responses (shown in pink) leading to the HR, while SHMT activity antagonizes GOX, repressing (blunt-ended lines) downstream defense responses. GOX, glycolate oxidase; SHMT, serine hydroxymethyltransferase; AGT, alanine glyoxylate transaminase; ET, ethylene; SA, salicylic acid; HR, hypersensitive response. Processes or genes found to be upregulated in the presence of avirulent pathogens or pathogen-derived elicitors are shown in yellow.

Sugar biosynthesis in plants occurs through the process of carbon fixation mediated by the enzyme ribulose-1,5-bisphosphate-carboxylase/oxygenase (RUBISCO). This enzyme catalyzes a carboxylase reaction that uses ribulose 1,5-bisphospate as a substrate and CO_2_ to produce two molecules of 3-phosphoglycerate (PGA) that are integrated into the Calvin cycle to form sugars (Peterhansel et al., 2010). The same enzyme catalyzes an oxygenase reaction converting ribulose 1,5-bisphosphate into 2-phosphoglycolate and 3-PGA during photorespiration. Further enzymatic activities using phosphoglycolate are essential as alternative pathways for synthesis of the amino acids glycine and serine (**Figure 2**). Therefore, photorespiration represents a converging point for carbohydrate and amino acid metabolic pathways. Recent studies have reported that, in addition to the physiological function as a salvage pathway for carbon loss, photorespiration is also involved in defense responses (Kangasjarvi et al., 2012).

One of the first enzymes in the photorespiratory pathway is glycolate oxidase (GOX) which catalyzes the conversion of glycolate into glyoxylate. This enzyme was produced in abundance in resistant somatic hybrids between *Brassica napus *and *Arabidopsis *in response to the fungal pathogen *Leptosphaeria maculans* infection (Bohman et al., 2002). In addition, *GOX* was also induced in barley upon inoculation with the pathogenic fungus *Bipolaris sorokiniana *(Schafer et al., 2004; **Figure 2**). We recently demonstrated that silencing of *GOX *in *N. benthamiana* delays the onset of the HR, and consequently plants become susceptible to nonhost pathogens *P. syringae *pv. tomato T1, *P. syringae *pv. glycinea and *Xanthomonas campestris *pv. vesicatoria (Rojas et al., 2012). Similarly, *Arabidopsis* *gox *mutants were susceptible to nonhost pathogens *P. syringae *pv. tabaci and *P. syringae *pv. syringae B728A, and showed a reduction in H_2_O_2_ accumulation and callose deposition (Rojas et al., 2012). Two of these mutants (*Atgox3 *and *Athaox2*) were further characterized and shown to have a considerable reduction in the expression of defense-related genes associated with the SA and ethylene (ET) pathways (Rojas et al., 2012; **Figure 2**).

As mentioned above, GOX reaction produces glyoxylate that can be further converted into glycine by two glyoxylate aminotransferases. These aminotransferases were found to have higher expression and enzymatic activities in melon cultivars that are naturally resistant to the oomycete pathogen *Pseudoperonospora cubensis *in comparison with susceptible cultivars (Taler et al., 2004). Interestingly, GOX activity in these resistant cultivars was ~10–20 times higher than susceptible cultivars. Because GOX is located upstream of the aminotransferases, it was proposed that the high aminotransferase activity in resistant cultivars causes a higher demand for glyoxylate, which in turn causes higher GOX activity. Therefore, resistance was not directly attributed to glyoxylate aminotransferases, but was attributed to the activity of GOX that releases H_2_O_2_ (Taler et al., 2004).

The gene encoding the mitochondrial enzyme serine hydroxymethyltransferase (*SHMT1*) that catalyzes the conversion of serine to glycine during photorespiration was implicated in altering HR when *Arabidopsis *plants were inoculated with the avirulent *P. syringae *pv. tomato DC3000 (*AvrRpm1*; Moreno et al., 2005; **Figure 2**). The *Arabidopsis* *shmt1-1 *mutant developed spontaneous lesion formation consistent with a constitutive expression of SA-responsive genes such as *PR-1*, *PR-2*, and *PR-5 *(Moreno et al., 2005; **Figure 2**). In wild-type *Arabidopsis* plants, *SHMT1* transcripts accumulated in response to pathogens such as *P. syringae *pv. tomato DC3000 (*AvrRpm1*) and *Alternaria brassicicola*. *PDF1.2* (*plant defensin 1.*2) was induced, one day after infection with *A. brassicicola*, in both wild-type and the *shmt1-1 *mutant, and the transcript induction was still observed four days after inoculation in wild-type plants, while it quickly declined in the *shmt1-1 *mutant and disappeared two days after inoculation (Moreno et al., 2005; **Figure 2**).

### AMINO ACID METABOLISM

Literature using transcriptomics in *Arabidopsis* revealed the upregulation of several genes involved in amino acid biosynthesis or homeostasis in response to avirulent *P. syringae *pv. tomato (*AvrRpt2*; Scheideler et al., 2002), *P. syringae *pv. tomato (*AvrRpm1*) and *P. syringae *pv. tomato (Hrc^-^; Less et al., 2011), or the pathogen-derived elicitors Flg22 and HrpZ (Less et al., 2011). Metabolite profiling in *Arabidopsis* also revealed that, while some amino acids such as valine, leucine, and tyrosine accumulate after treatment with avirulent and virulent pathogens, other amino acids are differentially accumulated depending upon the type of pathogen used (Ward et al., 2010). For example, inoculation with an avirulent strain of *P. syringae *pv. tomato DC3000 (*hrp^-^*mutant) revealed increased accumulation of tryptophan, tyrosine, lysine, valine, and leucine, and a decrease in glutamate in comparison with mock-inoculated plants, while inoculation with the virulent strain caused higher accumulation of isoleucine, threonine, alanine, phenylalanine, tyrosine, and glutamine in comparison with the accumulation of the same amino acids after inoculation with the avirulent strain. Other amino acids such as aspartate were reduced in both *P. syringae *pv. tomato DC3000 and *hrp^-^* mutant-treated plants (Ward et al., 2010). Although this study was conducted to understand how pathogens reconfigure their host plant metabolism, the significance of the study from the perspective of plant defense responses is not known. It is likely that particular amino acids, especially those that are accumulated or reduced regardless of the pathogen used, are involved in plant defense. However, this is just a speculation and more comprehensive studies are needed to prove this.

**FIGURE 3:**
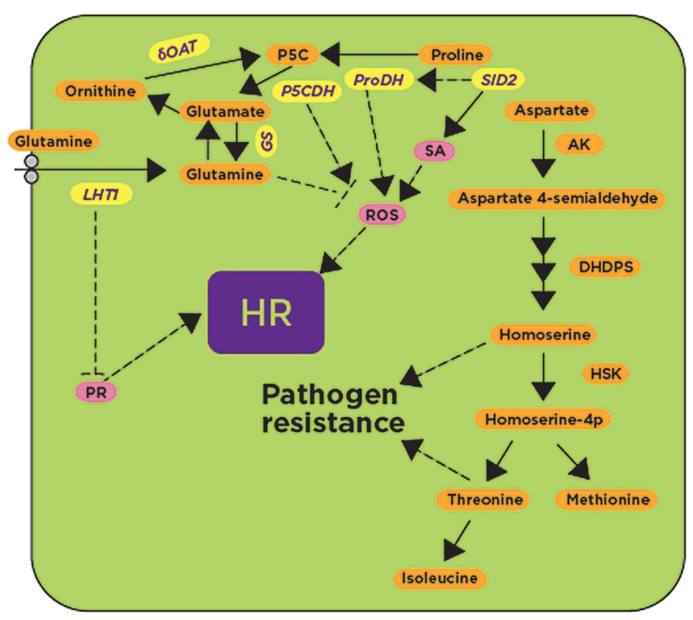
**Glutamine, proline, and aspartate metabolic pathways have been implicated in defense responses.** Genes involved in glutamine, proline and aspartate metabolism such as P5CDH, ProDH, and δOAT, as well as the glutamine transporter LHT1, have been shown to be induced after exposure to avirulent pathogens and pathogen-derived elicitors (shown in yellow). Their activities likely regulate known downstream defense responses such as the activation of pathogenesis-related proteins (PR), the generation of reactive oxygen species (ROS), and the synthesis of the hormone salicylic acid (SA; shown in pink) leading to the HR. Accumulation of aspartate-derived metabolites confers pathogen resistance by unknown mechanism(s). *SID2, salicylic acid deficient 2*; P5CDH, δ1-Pyrroline-5-carboxylate dehydrogenase; ProDH, proline dehydrogenase; δOAT, δ-ornithine aminotransferase; GS, glutamine synthase; LHT, lysine histidine transporter 1; AK, aspartate kinase; DHDPS, dihydrodipicolinate synthase 2; HSK, homoserine, kinase; HR, hypersensitive response. Dashed lines represent hypothetical regulatory mechanisms with arrows indicating positive regulation, while blunt-ended lines indicate negative regulation.

The aforementioned transcriptomics and metabolomics approaches suggested a role for amino acids in defense responses. However, more insightful studies came from mutants affected in amino acid metabolism. For example, the *lht1 *(*lysine histidine transporter 1*) mutant of *Arabidopsis*, that has significantly reduced contents of glutamine, alanine, and proline in comparison with wild-type plants, showed enhanced resistance to diverse bacterial, fungal, and oomycete pathogens such as *P. syringae *pv. tomato (*AvrRpt2*; avirulent), *P. syringae *pv. tomato DC3000 (virulent), *Colletotrichum higginsianum*, and *Eryshiphe cichoracearum *(Liu et al., 2010). After inoculation with these pathogens, the *lht1 *mutant supported less pathogen growth in comparison with wild-type plants. The *lht1 *mutant also exhibited increased callose deposition, higher accumulation of SA and constitutive expression of *PR-1* (Liu et al., 2010). Inoculation with these pathogens induced the *Lht1 *gene in wild-type plants, but this expression was abolished in transgenic plants expressing the *NahG *gene that converts SA to inactive catechol and in SA biosynthetic or signaling-related mutants such as *pad4*, *sid2*, and *npr1*, but not in the jasmonic acid (*jar1-1) *or ET (*ein2-1*, *etr1-1*) signaling mutants, suggesting that *Lht1*-mediated resistance is dependent on the SA pathway (**Figure 3**). Interestingly, *lht1 *mutants expressing* NahG *or the double mutants *lht1 pad4, lht1 sid2 *and *lht1 npr1 *are as susceptible as their respective single SA mutants to *C. higginsianum* and *E. cichoracearum*, and showed reduced *PR-1* expression (Liu et al., 2010). The authors suggest that the reduction in glutamine accounts for the defense-related phenotypes as the mutant that specifically hyper-secretes glutamine from hydathodes (*gdu1-1D, glutamine dumper 1*) also showed spontaneous lesions, increased H_2_O_2_ and callose accumulation (Pilot et al., 2004). In contrast, the exogenous addition of glutamine inhibited H_2_O_2_ production (Liu et al., 2010). As shown with *LHT*, other genes involved in amino acid metabolism are presumably involved in defense responses through a mechanism that is dependent on the SA pathway.

In plants, proline dehydrogenase (ProDH) converts proline to P5C and later to glutamate by P5CDH (δ1-Pyrroline-5-carboxylate dehydrogenase). In *Arabidopsis*, accumulation of proline was observed to be associated with the onset of HR after inoculation with avirulent pathogens (Fabro et al., 2004; **Figure 3**). *Arabidopsis* *proDH *mutants have increased susceptibility to avirulent pathogens, and the expression of the *ProDH *gene was found to be dependent on SA since no *ProDH* expression was found in the *sid2 *mutant (Cecchini et al., 2011). Interestingly, *ProDH*-silenced *Arabidopsis* lines had reduced ROS and cell death when compared to non-silenced control plants, indicating that ProDH potentiates the accumulation of ROS (Cecchini et al., 2011; **Figure 3**). External application of proline on *Arabidopsis* plants produced HR-like cell death symptoms without any pathogen inoculation (Deuschle et al., 2004). P5C accumulation in plants is also known to induce several defense-related genes that are otherwise induced only during pathogen infection (Deuschle et al., 2004). Evidence from several studies showed that the P5C-induced cell death pathway may contribute to the HR induced during avirulent pathogen inoculation (Deuschle et al., 2004; Cecchini et al., 2011; Senthil-Kumar and Mysore, 2012; **Figure 3**). In addition to ProDH, another enzyme involved in proline metabolism is the ornithine delta aminotransferase (δOAT) that was also found to mediate ROS accumulation in mitochondria, leading to the HR during nonhost resistance (Senthil-Kumar and Mysore, 2012). *δOAT*- and *ProDH1-*silenced *N. benthamiana *plants showed delayed HR after co-infiltration of *Pto*-*AvrPto *and *Cf9-Avr9 *by *Agrobacterium tumefaciens*, as well as after inoculation with the HR-inducing nonhost pathogen *P. syringae *pv. tomato T1 (Senthil-Kumar and Mysore, 2012). Furthermore, silencing of *δOAT* or *ProDH1* or *P5CS* caused increased growth of the nonhost bacterial pathogen *P. syringae *pv. tomato T1 when compared to non-silenced control plants (Senthil-Kumar and Mysore, 2012). *δOAT*, *P5CS *and *ProDH* were strongly induced in *N. benthamiana *at the site of inoculation with the nonhost pathogen *P. syringae *pv. tomato T1, but not induced by the host pathogen *P. syringae *pv. tabaci (Senthil-Kumar and Mysore, 2012). In agreement with the results obtained in *N. benthamiana, Arabidopsis* mutants containing T-DNA insertions in *AtProDH *and *AtδOAT *supported more bacterial growth and showed disease symptoms after inoculation with an *Arabidopsis* nonhost pathogen, *P. syringae *pv. tabaci (Senthil-Kumar and Mysore, 2012).

The amino acid homoserine is the precursor of other amino acids such as threonine, isoleucine, and methionine, and is synthesized from L-aspartate semialdehyde and further converted into O-phospho-L-homoserine by homoserine kinase. Mutation in the homoserine kinase encoded by *DMR1 *(downy mildew resistant 1) caused accumulation of the amino acid homoserine and increased resistance against the oomycete *H. arabidopsidis *by an unknown mechanism that is independent of the major defense signaling pathways such as SA, ET and jasmonic acid (JA; van Damme et al., 2009). Similarly, mutations in genes encoding *DHDPS2 *(dihydrodipicolinate synthase 2) and *AK2 *(aspartate kinase 2) showed increased resistance to *H. arabidopsidis *and increased accumulation of threonine, methione, and isoleucine in comparison with wild-type plants (Stuttmann et al., 2011; **Figure 3**). Threonine was pinpointed as the amino acid responsible for conferring resistance to *H. arabidopsidis*, but not to the fungal pathogen *Galovinomyces orontii*, presumably by altering the pathogen’s ability to grow under that condition. The same effect was observed in wild-type plants after exogenous application of threonine or by supplying threonine to synthetic media used for plant growth (Stuttmann et al., 2011). Like the *dmr1 *mutant, the resistance response to *H. arabidopsidis *in *dhdps2 *and *ak2 *mutants was independent of known defense pathways (**Figure 3**; Stuttmann et al., 2011). The accumulated evidence so far suggests that the accumulation of some amino acids or their metabolic byproducts triggers resistance responses against pathogens that can be dependent or independent of SA- and ROS-mediated defense pathways (**Figure 3**).

### LIPID METABOLISM

Lipids constitute a broad group of naturally occurring molecules with diverse biological functions; they provide structural components for the cell wall (in the form of waxes and cutin) and cell membrane, and also provide energy for metabolism (Welti et al., 2007). They are also mediators in many plant processes, including signal transduction, cytoskeletal rearrangements, and membrane trafficking (Wang, 2004). These processes are crucial for cell survival, growth and differentiation, and for plant responses to environmental cues, including biotic stress (Welti et al., 2007). In the current review, we discuss evidence supporting the role of lipid biosynthetic genes in defense responses. The activities of these enzymes regulate the spatial and temporal production of lipid metabolites that mediate responses to specific biotic cues (Wang, 2004; Zhao et al., 2013).

#### Biosynthesis of fatty acids

**FIGURE 4:**
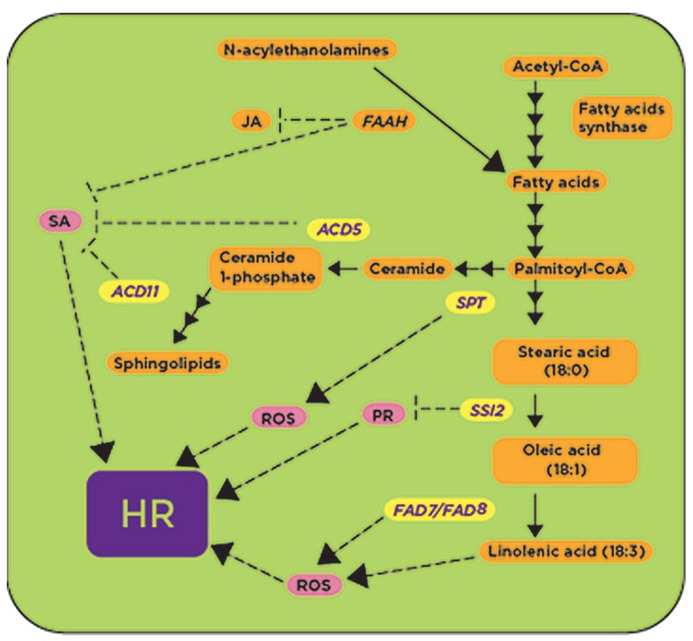
**Metabolic enzymes involved in fatty acid and sphingolipids biosynthetic pathways are involved in defense responses.** Some genes required for fatty acid and sphingolipid biosynthesis have been shown to be upregulated (shown in yellow) in the presence of avirulent pathogens and pathogen-derived elicitors. The activities of *ACD5*, *ACD11*, and *SSI2 *are proposed to play a negative role (shown with blunt-ended lines) regulating downstream defense responses such as the accumulation of reactive oxygen species (ROS), the expression of pathogenesis related (*PR*) genes and the synthesis of salicylic acid (SA; shown in pink), while the activities of *SPT *and *FAD7/FAD8 *likely induce (shown with arrows) defense responses. *ACD5*, accelerated cell death 5; *ACD11*, accelerated cell death 11; *FAAH: fatty acid hydrolase*; *SSI2*, suppressor of SA-insensitivity 2; *FAD7/FAD8*: fatty acid desaturases 7 and 8; *SPT*, serine palmitoyl transferase; HR, hypersensitive response.

One of the key steps in fatty acid biosynthesis is the desaturation of the stearic acid (18:0) to oleic acid (18:1) catalyzed by the stearoyl- desaturase encoded by the *SSI2/FAB2 *(suppressor of SA-insensitivity) gene (Lightner et al., 1994; Shah et al., 2001). The *Arabidopsis* *ssi2 *mutant was isolated as a suppressor of *npr1-5* mutation (Kachroo et al., 2001), which fails to activate *PR *gene expression after SA treatment. The deficiency of SSI2 causes a high accumulation of 18:0 fatty acids and decreased levels of 18:1 fatty acids in the *ssi2 *mutant in comparison with the wild-type (Kachroo et al., 2001). The *ssi2 *mutant showed higher expression of the resistance (*R*) genes *RPM1*, *SNC1*, *SSI4*, *RPP1, *and *RPS2 *(Mandal et al., 2012), and exhibited spontaneous lesion formation associated with high levels of SA and constitutive expression of *PR-1*, *PR-2,* and *PR-5 *genes (**Figure 4**; Shah et al., 2001). As a consequence of this constitutive activation of defense responses, the *ssi2* mutant is resistant to the oomycete *H. arabidopsidis *strain Emco5 and the virulent bacterial strain *P. syringae *pv. maculicola (Nandi et al., 2003). Remarkably, a considerable number of mutations from SA and nitric oxide (NO) signaling pathways have been found to suppress *ssi2 *phenotypes (Kachroo et al., 2005; Mandal et al., 2012), indicating that SA and NO intersect with fatty acid metabolic pathways through *SSI2*.

Another important step in fatty acid biosynthesis is the desaturation of fatty acids mediated by fatty acid desaturase 7 (*FAD7*) and fatty acid desaturase 8 (*FAD8*), and these genes have been linked to defense responses (Yaeno et al., 2004). In *Arabidopsis*, the double mutant *fad7/fad8* had low trienoic fatty acid (TA; 16- and 18-carbon fatty acids with three cis double bonds) content and reduced O_2_^-^ and H_2_O_2_ accumulation after inoculation with *P. syringae* pv. *tomato* DC3000 (*AvrRpm1*; Yaeno et al., 2004; **Figure 4**). Since O_2_^-^ and H_2_O_2_ are required for the onset of the HR, this *fad7/fad8 *double mutant also had reduced cell death when compared with wild-type and was unable to suppress the growth of the *P. syringae *pv. tomato DC3000 (*AvrRpm1*) or *P. syringae *pv. tomato DC3000 (*AvrRpt2*; Yaeno et al., 2004). All these phenotypes were specifically due to the defect in the biosynthesis of linolenic acid (LA) in the *fad7/fad8 *double mutant, as LA was shown to activate the O_2_^-^-generating enzyme NADPH oxidase (Yaeno et al., 2004; **Figure 4**).

Fatty acids can also be derived from *N-*acylethanolamines (NAEs), and exogenous application of NAEs induces the expression of defense-related genes (Tripathy et al., 1999). NAEs are hydrolyzed into fatty acid and ethanolamine by the fatty acid hydrolase (FAAH). Interestingly, *FAAH *overexpression compromised plant immunity against nonhost and host pathogens in *Arabidopsis* (Kang et al., 2008). *AtFAAH *overexpressor lines developed disease symptoms faster and accumulated more bacteria than the wild-type Col-0 plants after inoculation with nonhost pathogens *P. syringae *pv. syringae and *P. syringae *pv. tabaci, as well as host pathogens *P. syringae *pv. tomato DC3000 and *P. syringae *pv. maculicola (Kang et al., 2008). *AtFAAH *overexpressors showed reduced accumulation of SA and JA after mock and pathogen inoculation while the levels of abscisic acid (ABA) were only reduced in wild-type Col-0 after inoculation with a nonhost pathogen (Kang et al., 2008). Transcripts for genes involved in SA-mediated defense signaling, as well as some transcripts from JA-mediated signaling, were less abundant in the *AtFAAH *overexpressor (Kang et al., 2008; **Figure 4**). Together, all these data indicate that, as a whole, fatty acid metabolism represents a convergence point for defense signaling cascades involving SA, JA and NO (**Figure 4**).

#### Biosynthesis of sphingolipids

Sphingolipids have been shown to trigger the process of PCD through the generation of ROS in connection with the fungal toxins AAL (named after the pathogen *alternata alternata* that produces this toxin) and fumonisin (from *Fusarium sp*; Berkey et al., 2012). The sphingoid portion or long-chain base (LCB) of the sphingolipids is synthesized by the activity of the heterodimeric enzyme serine palmitoyltransferase (SPT). LCB1, a subunit of SPT, is the target of the apoptotic inducer fumonisin B1 (FB). The *Arabidopsis* *lcb1 *mutant is resistant to FB and hence incapable of generating ROS and initiating PCD after FB treatment. FB-dependent cell death was found to be influenced by ROS production and by high levels of sphingoid bases (Shi et al., 2007). The LCB2 subunit of SPT1 was found to be induced in potato after infection with *P. infestans *(Birch et al., 1999). The expression of *NbLCB2*, driven by the dexamethasone inducible promoter, caused spontaneous cell death in *N. benthamiana *after dexamethasone treatment (Takahashi et al., 2009). In addition, using VIGS, it was demonstrated that downregulation of *NbLCB1*and* NbLCB2 *allowed higher growth of the nonhost pathogen *P. cichorii*, while having no effect on the host pathogen *P. syringae *pv. tabaci (Takahashi et al., 2009).

Other genes involved in sphingolipid biosynthesis, shown to be involved in PCD-mediated defense responses in *Arabidopsis*, and induced by virulent and avirulent strains of *P. syringae* are *Accelerated Cell Death 5 *(*ACD5*) and *ACD11*, which encode ceramide kinase (CERK) and sphingoside transfer protein, respectively (Brodersen et al., 2002; Liang et al., 2003). Both *acd5 *and *acd11 Arabidopsis* mutants showed spontaneous cell death and exhibited typical features of animal apoptosis (Greenberg et al., 2000; Brodersen et al., 2002). Intriguingly, in spite of increased accumulation of SA and the phytoalexin camalexin in the *acd5 *mutant, disease symptoms caused by the virulent pathogen *P. syringae *pv. tomato DC3000 and its growth were enhanced when compared to wild-type plants (Greenberg et al., 2000). *ACD5 *was induced by both avirulent *P. syringae* pv. tomato (*AvrRpm1*) as well as virulent *P. syringae *pv. tomato DC3000, and this induction occurred early after inoculation with the avirulent pathogen (Liang et al., 2003). Gene expression profiling of the *acd11 *mutant in comparison with wild-type plants revealed strong upregulation of genes involved in oxidative burst, PCD and defense signal transduction pathways (Brodersen et al., 2002). Further genetic analysis in *acd11/pad4-2*, *acd11/eds1-2* and *acd11/sid2-2 *double mutants indicated that *acd11 *phenotypes are SA-dependent (Brodersen et al., 2002, 2005; **Figure 4**), requiring *PAD4* and *EDS1*. In contrast, genetic analysis of *acd11/ein2-1 *and *acd11/jar1-1 *double mutants indicated that *acd11 *phenotypes are independent of JA and ET (Brodersen et al., 2002). All these data indicate that perturbation of sphingolipid metabolism leads to SA-dependent PCD, most likely associated with accumulation of sphingoids and ceramides, although the exact molecule(s) triggering these responses is not known (**Figure 4**).

## CONCLUSION

The complexity of plant defense responses requires an abundant supply of energy, primarily derived from primary metabolic processes (Bolton, 2009). In this review, we discussed how plants use primary metabolic pathways not only as a source of energy to drive extensive defense responses, but also as a source of signaling molecules to directly or indirectly trigger defense responses. Many genes involved in carbohydrate catabolism processes such as glycolysis and the TCA cycle are upregulated during plant defense responses, although only *cwINV* (Herbers et al., 1996a), *HXK *(Xiao et al., 2000), and *PDC* (Tadege et al., 1998) have been shown to induce defense responses such as the activation of *PR *genes leading to the onset of HR. Although downregulation of photosynthesis during biotic stress responses has been reported, the expression of RUBISCO, a key enzyme for carbon fixation events in the Calvin cycle, is upregulated (Less et al., 2011). It is not clear from the literature whether the upregulation of RUBISCO is to provide more sugars to create a defense-positive amplification loop or whether this upregulation favors the oxygenase reaction leading to photorespiration. The intrinsic connection between carbohydrate metabolism, photorespiration and biosynthesis of some amino acids adds additional components for defense by the generation of H_2_O_2_ by GOX (Rojas et al., 2012) and the activation of SA-mediated pathways by proline and proline-derived metabolites (Fabro et al., 2004; Deuschle et al., 2004; Cecchini et al., 2011; Senthil-Kumar and Mysore, 2012).

In addition to proline, other amino acids are also likely to influence the outcome of plant-pathogen interactions, but have not been studied in detail. Transcriptomics and metabolomics data indicate differential gene expression or accumulation of various amino acids, depending on the stimuli (Scheideler et al., 2002; Ward et al., 2010). However, the lack of functional characterization of these differentially expressed genes or metabolites makes it difficult to interpret the significance of these findings. More work is needed to understand why some amino acids are differentially accumulated and what the impact of such response is.

The defense signaling cascade mediated by carbohydrate metabolism, photorespiration and amino acid metabolism is presumably negatively regulated when no longer needed. Several genes, such as *CPR5 *(Bowling et al., 1997), *SHMT *(Moreno et al., 2005), and *LHT1 *(Liu et al., 2010), have already been identified as playing a negative role during normal conditions. In addition to carbohydrate and amino acid metabolism, the role of lipids and lipid metabolism in defense responses has been comprehensively studied, especially genes associated with biosynthesis of fatty acids and sphingolipids (Kachroo and Kachroo, 2009; Berkey et al., 2012). Several genes from these pathways were shown to be induced by avirulent pathogens and elicitors. In addition, accumulation of a particular family of lipids such as sphingolipids, phospholipids or linolenic acid triggers ROS accumulation followed by the onset of the HR during plant defense responses.

## FUTURE PERSPECTIVES

In this review, we compiled evidence to show that the components of primary metabolic pathways are directly or indirectly involved in the induction of a plethora of defense responses aimed at preventing or stopping the proliferation of a potential pathogen. The combination of activities from primary metabolic pathways appears to be highly redundant, which explains in part the energy cost for the plant (Bolton, 2009). In spite of substantial evidence of the role of primary metabolism in plant defense responses, still more studies are needed to identify additional components involved in defense responses as well as detailed characterization of the mechanisms underlying such responses. To our knowledge, there has not been a corresponding effort toward engineering primary metabolism to achieve disease resistance in agronomically important crops. It is possible that fine-tuning of primary metabolic pathways by modifying activity or expression of key enzymes might be enough to achieve disease resistance without compromising crop yield. Because primary metabolism is essential for plant growth and development, genes involved in such processes are less likely to be eliminated by plant natural selection as is the case for *R*-genes; therefore, engineering resistance against pathogens by manipulating primary metabolic pathways is expected to be more durable.

## Conflict of Interest Statement

The authors declare that the research was conducted in the absence of any commercial or financial relationships that could be construed as a potential conflict of interest.
